# Evaluation of a New Telemedicine System for Early Detection of Cardiac Instability in Patients With Chronic Heart Failure: Real-Life Out-of-Hospital Study

**DOI:** 10.2196/52648

**Published:** 2024-08-13

**Authors:** Jean Marie Urien, Emmanuelle Berthelot, Pierre Raphael, Thomas Moine, Marie Emilie Lopes, Patrick Assayag, Patrick Jourdain

**Affiliations:** 1 CHU Bicêtre Le Kremlin Bicêtre France; 2 Institut du cœur saint Gatien Tours France; 3 Clinique Saint Gatien Tours France

**Keywords:** telemedicine system, follow-up, detection, heart failure, chronic heart failure, CHF, heart disease, ambulatory patient, ambulatory patients, home-based, TwoCan Pulse, telecardiology, cardiology, e-device, mHealth, mobile health, app, apps, application, applications, effectiveness, real-life setting, remote monitoring, virtual monitoring, France, men, gerontology, geriatric, geriatrics, older adult, older adults, elder, elderly, older man, ageing, aging

## Abstract

**Background:**

For a decade, despite results from many studies, telemedicine systems have suffered from a lack of recommendations for chronic heart failure (CHF) care because of variable study results. Another limitation is the hospital-based architecture of most telemedicine systems. Some systems use an algorithm based on daily weight, transcutaneous oxygen measurement, and heart rate to detect and treat acute heart failure (AHF) in patients with CHF as early on as possible.

**Objective:**

The aim of this study is to determine the efficacy of a telemonitoring system in detecting clinical destabilization in real-life settings (out-of-hospital management) without generating too many false positive alerts.

**Methods:**

All patients self-monitoring at home using the system after a congestive AHF event treated at a cardiology clinic in France between March 2020 and March 2021 with at least 75% compliance on daily measurements were included retrospectively. New-onset AHF was defined by the presence of at least 1 of the following criteria: transcutaneous oxygen saturation loss, defined as a transcutaneous oxygen measurement under 90%; rise of cardiac frequency above 110 beats per minute; weight gain of at least 2 kg; and symptoms of congestive AHF, described over the phone. An AHF alert was generated when the criteria reached our definition of new-onset acute congestive heart failure (HF).

**Results:**

A total of 111 consecutive patients (n=70 men) with a median age of 76.60 (IQR 69.5-83.4) years receiving the telemonitoring system were included. Thirty-nine patients (35.1%) reached the HF warning level, and 28 patients (25%) had confirmed HF destabilization during follow-up. No patient had AHF without being detected by the telemonitoring system. Among incorrect AHF alerts (n=11), 5 patients (45%) had taken inaccurate measurements, 3 patients (27%) had supraventricular arrhythmia, 1 patient (9%) had a pulmonary bacterial infection, and 1 patient (9%) contracted COVID-19. A weight gain of at least 2 kg within 4 days was significantly associated with a correct AHF alert (*P*=.004), and a heart rate of more than 110 beats per minute was more significantly associated with an incorrect AHF alert (*P*=.007).

**Conclusions:**

This single-center study highlighted the efficacy of the telemedicine system in detecting and quickly treating cardiac instability complicating the course of CHF by detecting new-onset AHF as well as supraventricular arrhythmia, thus helping cardiologists provide better follow-up to ambulatory patients.

## Introduction

Chronic heart failure (CHF) remains a significant public health challenge, characterized by frequent destabilization leading to hospitalizations, decreased quality of life, and increased mortality rates [1]. Over the past decade, despite numerous studies exploring telemedicine systems, there has remained a dearth of consensus on recommendations for CHF care due to inconsistent findings [2]. Moreover, most existing telemedicine systems are hampered by their hospital-based architecture, limiting their effectiveness in real-world, out-of-hospital settings [3].

To address these limitations, some systems offer a novel approach to CHF management. Using a proprietary algorithm integrating daily weight, transcutaneous oxygen measurement (TCOM), and heart rate, these systems aim to detect and treat acute heart failure (AHF) episodes in patients with CHF at the earliest stage. By facilitating early intervention, this system promises to reduce the burden of hospitalization and improve patient outcomes [4-6].

Despite its potential, there remains a need to evaluate the efficacy of these kinds of systems in real-life settings. Specifically, it is crucial to assess their ability to detect clinical destabilization in out-of-hospital environments while minimizing false positive alerts. Therefore, the primary objective of this study is to investigate the performance of one such device in detecting AHF episodes and supraventricular arrhythmias in patients with CHF undergoing out-of-hospital monitoring.

In this retrospective analysis, we present findings from a cohort of patients with CHF who underwent self-monitoring at home using the telemonitoring system following a congestive AHF event. By analyzing data collected over a 1-year period, we aim to elucidate the system’s ability to accurately identify AHF episodes and its impact on clinical outcomes. Additionally, we explore factors associated with both correct and incorrect AHF alerts, shedding light on the system’s strengths and limitations in real-world use.

Through this investigation, we seek to provide valuable insights into the role of telemedicine systems in enhancing the management of patients with CHF in ambulatory settings. By demonstrating their effectiveness in early detection and intervention, we aim to support the integration of such technologies into routine clinical practice, thereby improving the care continuum for patients with CHF.

## Methods

### Study Population

This study was a retrospective single-center observational cohort study of patients hospitalized with CHF during the previous 3 months at high risk of further AHF who were included in the Expérimentations de Télémédecine pour l’Amélioration des Parcours En Santé (ETAPES) national telemedicine experimentation program and were provided with the telemonitoring system upon discharge to home from the Nouvelle Clinique Tourangelle of Tours, France, regardless of age, sex, social status, or left ventricular ejection fraction (LVEF) at inclusion.

All consecutive patients using the telemonitoring system (TwoCan Pulse) for early detection of AHF and presenting sufficient compliance with a follow-up of at least 1 year with the TwoCan Pulse between March 1, 2020, and March 1, 2021, were included. As required by the ETAPES program, inclusion criteria were clinical decompensation of heart failure (HF) in the last 12 months plus symptomatic HF with New York Heart Association class II or above and a brain natriuretic peptide level greater than 100 pg/mL. Patients were excluded who dropped out during follow-up because of telephone network difficulty or discomfort; had insufficient compliance, defined as daily measurements during less than 75% of the study period; had uncontrolled supraventricular arrhythmia, particularly if the heart rate was above 100 beats per minute (bpm); had an unstable state, ruling out home discharge; or had a life expectancy under 1 year.

### CHF, Telemonitoring System Compliance, and Education

CHF was defined according to the European Society of Cardiology 2021 Heart Failure Guidelines criteria [2], including HF with reduced ejection fraction (HFrEF) and HF with preserved ejection fraction (HFpEF). Duration of HF was arbitrarily divided into new-onset HF if it was more recent than 1 year and long-duration HF if longer than 1 year.

Sufficient compliance was defined as daily use of the telemonitoring system at least 75% of the time in the defined period of this study. Every hospitalized patient with HF meeting the criteria was offered a TwoCan Pulse device before being discharged to home. Patients who accepted the offer were instructed on use of the device and received personalized therapeutic and educational support from trained nurses. Only 5% of screened patients turned down the offer and were excluded from the study.

### HF Destabilization Alert

Weight, TCOM taken on a finger, and cardiac frequency were self-monitored by the patients once daily. The data were sent instantly by Bluetooth transmitter to a secured website, and a medical team composed of a nurse and 2 cardiologists monitored the patients’ status and alerts 6 days a week.

New-onset AHF alert criteria were based on a predefined clinical algorithm involving the presence of at least 1 criterion among the following: transcutaneous oxygen saturation loss defined as TCOM under 90%, rise of cardiac frequency above 110 bpm, and weight gain of at least 4 kg; or the presence of at least 2 criteria among the following: transcutaneous oxygen saturation loss defined as TCOM under 92%, rise of cardiac frequency to above 90 bpm, and weight gain of at least 2 kg within 4 days. Stable patients were defined as patients without any of these criteria. The alarm triggered if 1 of the following 4 conditions was reached: heart rate above 110 bpm, oxygen saturation <90%, weight gain >4 kg compared to baseline weight, and weight gain of >4 kg in 4 days. The alarm was also triggered if 2 of the following conditions were met: heart rate was above 90 bpm and below 110 bpm, oxygen saturation <92% and ≥90%, and weight gain >2 kg in 2 days. The alarm was sent to a small central device provided to the patient and to the cardiologist, either via email, if they preferred, or on the TwoCan server. To our knowledge, no other remote monitoring system uses an algorithm that takes into account these different values. For compliance measurement, patients were included if they recorded at least 75% of their 3 values each day.

In order to encourage patient engagement and self-care, the patients were informed of their status by a green light in the absence of any AHF alert and a red light and an alarm in the event of an AHF alert.

In the event of an alert signal on their device, patients were to call their cardiologist within 72 hours to assess the need for diuretic treatment adjustment, an emergency doctor’s appointment or, if indicated, admission to the cardiology unit. If patients did not call their cardiologist as expected, the medical team called them within 48 hours. In the absence of any referring cardiologist, patients were to call the emergency number of their referring center, allowing for an answer from a cardiologist 7 days a week. Every alert was checked over the phone by a nurse or a cardiologist, based on breathlessness symptom evaluation or onset of edema, and was classified retrospectively as AHF if the patient’s state stabilized after diuretic dose adjustment.

### Baseline Assessment and Follow-Up

Baseline data, including demographics, cardiomyopathy characteristics, treatment plan, chronic renal impairment, and/or chronic obstructive pulmonary disease, as well as LVEF at inclusion, were collected from hospital medical charts for all enrolled patients. The collected in-hospital data included the presence and type of alert (weight gain, transcutaneous oxygen loss, or excessive heart rate), and clinical response (call, emergency doctor’s appointment, and/or hospitalization). Every patient received a standard follow-up visit at 1 year after inclusion, whether there was any AHF alert or not, to check on any AHF occurrence since inclusion that may not have been detected by the TwoCan Pulse.

### Study Aims

The aim of the study was to analyze patient phenotypes, rate and types of alerts, and the efficacy of the telemonitoring system algorithm in detecting any destabilization of HF in outpatients with CHF.

### Statistical Analysis

Qualitative variables were expressed as numbers (percentages) and continuous data as means (SDs) or medians (IQRs), depending on their distribution. The *t* test (2-tailed) was used for continuous variables and the *χ*^2^ test for comparing percentages. *P*<.005 was considered significant. Survival rates were summarized using Kaplan-Meier estimates, and log-rank tests were used to compare groups. All tests were 2-sided at a significance level of .05. Statistical analyses were conducted using R++ (Zebrys).

### Ethical Considerations

The study was compliant with Helsinki rules and was approved by the local ethics committee (Commission éthique et déontologie de la Faculté de Médecine Paris-Saclay; 20181128163709). The Scientific and Ethical Committee of the Health Data Warehouse of AP-HP approved the protocol (EDS CSE 180032). Informed consent was obtained for all the participants. Statements regarding human subject research ethics review, exemptions, and approvals were obtained. This study was part of research involving human subjects and is subject to rigorous regulation in France. Anonymization in studies involving human subjects in France involves removing or coding directly identifiable personal data, aggregating data where possible, generalizing specific details to prevent identification, and having anonymized data reviewed by an ethics committee before publication. Compensation for participation in studies included reimbursement for travel expenses, compensation for time spent participating, or provision of medical care or services related to the study. There is no identification of individual participants or users in any images in this paper or its supplementary material.

## Results

### Study Population

Between March 1, 2020, and March 1, 2021, a total of 111 consecutive patients were included in the study. Baseline characteristics of these patients are presented in Table 1. In summary, 63% (n=70) were male, and the median age was 76.6 (IQR 69.49-83.41) years. The underlying cardiomyopathy was coronary artery disease in 53 patients (47.7%) and primary dilated cardiomyopathy in 30 patients (27%). Forty-four patients (39.6%) had a history of atrial fibrillation. An underlying HF type with reduced LVEF was found in 82 patients (73.8%), with an optimal underlying treatment in HFrEF in 57 (55.3%) of those patients and a median duration of HF of 1179 (IQR 61.5-4391.5) days.

**Table 1:** Population characteristics of patients included in the study who used the device.

Characteristics		Overall patients (N=111)	Patients with AHF^a^ alert (n=39)	Patients without AHF alert (n=72)	*P* value	
AHF within a year after inclusion, n		28	28	0	<.001	
Age (years), median (IQR)		76.60 (69.49-83.41)	76.93 (69.71-82.86)	75.3 (69.06-83.73)	.92	
Male, n (%)		70 (63)	29 (74.3)	41 (56.9)	.10	
Weight (kg), median (IQR)		75.00 (64.00-89.00)	72.5 (61.75-80.25)	79 (72-96)	.02	
**Cardiovascular risk factors, n (%)**						
	Hypertension	50 (45.1)	33 (45.8)	17 (43.6)	.84	
	Diabetes mellitus	22 (19.8)	10 (13.9)	12 (30.8)	.05	
	Dyslipidemia	77 (69.4)	51 (70.8)	26 (66.7)	.67	
	History of smoking	23 (20.7)	15 (20.8)	8 (20.5)	>.99	
	Family history of CVD^b^	3 (2.7)	3 (4.2)	0 (0)	.55	
**Cardiomyopathy, n (%)**						.71
	CAD^c^	53 (47.7)	36 (50)	17 (43.5)		
	Dilated cardiomyopathy	30 (27)	20 (27.5)	10 (27.6)		
	Hypertrophic cardiomyopathy	4 (3.6)	2 (2.7)	2 (5.1)		
	Hypertensive cardiopathy	7 (6.3)	7 (9.7)	0 (0)		
	Aortic valvular stenosis	7 (6.3)	2 (2.7)	5 (12.8)		
	CAD plus aortic valvular stenosis	3 (2.7)	0 (0)	3 (7.6)		
	CAD plus mitral regurgitation	2 (1.8)	2 (2.7)	0 (0)		
	Valvular prosthesis	1 (0.9)	1 (1.3)	0 (0)		
	Amylosis	4 (3.6)	2 (2.7)	2 (5.1)		
**Duration of heart failure**						
	Duration (days), median (IQR)	1179 (61.5-4391.5)	706 (23.75-2359.75)	804 (24-2897.5)	.14	
	New onset of HF^d^, n (%)	49 (44.1)	15 (38.4)	(47.2)	.43	
	HF lasting for at least 1 year, n (%)	62 (56.9)	24 (61.6)	38 (52.8)	.10	
	History of atrial fibrillation, n (%)	44 (39.64)	11 (28.2)	33 (45.8)	>.99	
	Chronic renal insufficiency, n (%)	10 (9.01)	8.00 (11.1)	2 (5.1)	.49	
	Chronic obstructive pulmonary disease, n (%)	3 (2.7)	2.00 (2.8)	1 (2.6)	.55	
**Treatment, n (%)**						>.99
	β-Blockers	99 (89.1)	35 (89.7)	64 (88.8)	.02	
	Angiotensin-converting-enzyme inhibitor or angiotensin II type I receptor blocker	66 (59.4)	17 (43.5)	47 (65.2)	>.99	
	Angiotensin receptor neprilysin inhibitor	17 (15.3)	6 (15.3)	11 (15.2)	>.99	
	Mineralocorticoid receptor antagonist	36 (32.4)	13 (33.3)	23 (31.9)	>.99	
	Ivabradine	3 (2.7)	1 (2.5)	2 (2.7)	.81	
	Furosemide	87 (78.4)	30 (76.9)	57 (79.1)	.12	
	Dose (mg/day)	104	104	93	.86	
	Optimal medical treatment in HFrEF,^e^ n (%)	57 (55.3)	16.0 (48.5)	41.0 (58.6)	>.99	
**Heart failure**						
	HFrEF, n (%)	82 (73.8)	29 (74.4)	53 (73.6)	.14	
	HFpEF^f^, n (%)	29 (25.2)	10 (25.6)	19 (26.4)	.25	
	Left ventricular ejection fraction at inclusion (%), median (IQR)	40.00 (31.50-50.00)	40 (30-48.12)	41 (35.5-50)	.14	

^a^AHF: acute heart failure.

^b^CVD: cardiovascular disease.

^c^CAD: coronary artery disease.

^d^HF: heart failure.

^e^HFrEF: heart failure with reduced ejection fraction.

^f^HFpEF: heart failure with preserved ejection fraction.

### Characteristics of Ambulatory Follow-Up

As detailed in Tables 1 and 2, in 1 year, 39 patients (35.1%) presented at least 1 AHF alert; of these alerts, 28 (72%) were confirmed AHF alerts and 11 (28%) were unconfirmed AHF alerts. No statistical differences were found between the confirmed AHF alert group and the unconfirmed AHF alert group in terms of medical characteristics, such as ejection fraction or age.

As summed up in Table 3, among unconfirmed AHF alerts, 5 patients (45%) had taken inaccurate measurements (especially for weight), 3 patients (27%) had supraventricular arrhythmia, 1 patient (9%) had a pulmonary bacterial infection, and 1 patient (9%) contracted COVID-19. Median time from inclusion to AHF alert was 100 (IQR 36.00-205) days, with a significant difference between the confirmed AHF alert group and the unconfirmed AHF alert group (150.5, IQR 72.5-254.5 days vs 75.3, IQR 69.06-83.73 days; *P*=.01).

As detailed in Table 4, the sensitivity of the telemonitoring system was 100%, and its specificity was 86.7%. The positive predictive value was 71.7%, and the negative predictive value was 100%.

To resolve AHF alerts, a medical consultation by phone sufficed for 22 patients (56%), an emergency medical appointment was required for 14 patients (36%), and hospitalization was required for 3 patients (8%). A weight gain of 4 kg or more within 4 days was significantly associated with a confirmed AHF alert (*P*=.004) and a resting heart rate faster than 110 bpm was more significantly associated with an unconfirmed AHF alert (*P*=.007). No patient had AHF in the absence of any AHF alert.

**Table 2:** Characteristics of patients with confirmed acute heart failure (HF) alert.

Characteristics		Patients with confirmed acute HF alert (n=28)	Patients without confirmed acute HF alert (n=83)	*P* value	
Age (years), median (IQR)		81.27 (72.41-88.56)	75.5 (69.04-82.48)	.05	
Male, n (%)		22 (79)	48 (58)	.07	
Weight (kg), median (IQR)		77 (67.5-92.25)	74 (62.5-87.5)	.33	
**Cardiovascular risk factors, n (%)**					
	Hypertension	14 (50)	36 (43)	.66	
	Diabetes mellitus	7 (25)	15 (18)	.42	
	Dyslipidemia	10 (36)	24 (29)	.49	
	History of smoking	5 (18)	18 (22)	.79	
	Family history of cardiovascular disease	3 (4)	0 (0)	.57	
**Cardiomyopathy, n (%)**					.21
	CAD^a^	11 (39)	42 (51)		
	Dilated cardiomyopathy	6 (21)	24 (29)		
	Hypertrophic cardiomyopathy	1 (4)	3 (4)		
	Hypertensive cardiopathy	2 (7)	5 (6)		
	AVS^b^	2 (7)	5 (6)		
	CAD plus AVS	4 (14)	2 (2)		
	Amylosis	2 (2)	2 (7)		
**HF characteristics**					
	Duration (days), median (IQR)	708.5 (78.75-4524.5)	883 (24-2816.5)	.31	
	New onset of HF, n (%)	11 (39)	38 (46)	.66	
	HF lasting for at least 1 year, n (%)	17 (61)	45 (54)	.13	
	History of atrial fibrillation, n (%)	21 (75)	46 (55)	.08	
	Chronic renal insufficiency, n (%)	2 (7)	8 (10)	>.99	
	Chronic obstructive pulmonary disease, n (%)	1 (4)	2 (2)	>.99	
**Treatment, n (%)**					
	β-Blockers	24 (86)	76 (92)	.46	
	Angiotensin-converting-enzyme inhibitor or angiotensin II type I receptor blocker	13 (46)	53 (64)	.12	
	Angiotensin receptor neprilysin inhibitor	9 (32)	27 (35)	>.99	
	Mineralocorticoid receptor antagonist	3 (11)	14 (17)	.55	
	Ivabradine	1 (4)	1 (1)	>.99	
	Furosemide	21 (75)	67 (81)	.59	
	Optimal medical treatment in HFrEF,^c^ n (%)	11 (39)	46 (55)	.19	
**Heart failure, n (%)**					.46
	HFrEF	19 (68)	63 (76)	.32	
	HFpEF^d^	9 (32)	20 (24)	.42	
Left ventricular ejection fraction at inclusion, median (IQR)		44 (39.25-51.25)	40 (30-47.25)	.06	

^a^CAD: coronary artery disease.

^b^AVS: aortic valvular stenosis.

^c^HFrEF: heart failure with reduced ejection fraction.

^d^HFpEF: heart failure with preserved ejection fraction.

**Table 3:** Acute heart failure (AHF) alert characteristics.

Characteristics		Total AHF alerts (n=39)	Confirmed AHF alerts (n=28)	Unconfirmed AHF alerts (n=11)	*P* value	
Days after inclusion, median (IQR)		100 (36.00-205)	150.5 (72.5-254.5)	49 (18-64)	.01	
**Duration of heart failure, n (%)**						>.99
	Heart failure lasting for at least 1 year	15 (38)	17 (61)	7 (64)	.35	
	New onset of chronic heart failure	24 (62)	11 (39)	4 (36)	.38	
**Type of AHF alert, n (%)**						
	Weight gain	25 (64)	21 (75)	4 (36)	.004	
	Loss of oxygen	5 (13)	3 (11)	2 (18)	.23	
	Increased heart rate	4 (10.2)	0 (0)	4 (36)	.02	
	Weight gain plus loss of oxygen	3 (7)	3 (11)	0 (0)	.99	
	Weight gain plus increased heart rate	0 (0)	0 (0)	0 (0)	.99	
	Loss of oxygen plus increased heart rate	1 (2.5)	0 (0)	1 (9)	.99	
	Weight gain plus loss of oxygen plus increased heart rate	1 (2)	1 (3)	0 (0)	.99	
**Medical response, n (%)**						
	Call	22 (56)	17 (60)	5 (45)	.54	
	Emergency consultation	14 (36)	7 (25)	7 (64)	.68	
	Hospitalization	3 (8)	3 (10)	0 (0)	.36	
**Type of unconfirmed AHF alert, n (%)**						
	Supraventricular arrhythmia	—^a^	—	3 (27)	—	
	Inaccurate measurements	—	—	5 (45)	—	
	Community-acquired bacterial pneumonia	—	—	1 (9)	—	
	COVID-19	—	—	1 (9)	—	

^a^Not applicable.

**Table 4:** Performance table of telemonitoring system.

	Global, %	Weight gain, %	Loss of oxygen, %	Increased heart rate, %
Sensitivity	100%	84	3.5	0
Specificity	86.7	94.7	96	98.6
Positive predictive value	71.7	84.6	60	0
Negative predictive value	100	83.7	74.2	65.5

## Discussion

### Principal Findings

The effectiveness of telemonitoring systems in detecting patients presenting with HF seems to have been established by the fact that no patient had AHF within a year of follow-up without being detected by the telemonitoring system algorithm.

Our algorithm, based on the analysis of 3 constants, appears to have been able to detect every episode of AHF in our study. It also detected other diseases requiring prompt care, such as community-acquired bacterial pneumonia and (supraventricular) arrhythmia, as proven by the isolated higher heart rate found in 3 patients with unconfirmed AHF alerts.

Therefore, the telemonitoring system may help cardiologists detect patients at risk of destabilization of their underlying cardiopathy beyond AHF and allow them to be treated as promptly as possible to avoid significant complications such as stroke in atrial fibrillation. This study is in line with many other studies [4,7-11] that have proven the efficacy of telemedicine systems in helping avoid unplanned hospitalizations and improving HF self-management by patients [12-14].

The main advantages of telemonitoring systems are simplicity of data acquisition and ease of handling for patients, making such systems broadly available to all patients, even in old age. In fact, telemonitoring systems only require a phone connection to send daily measurements. A team of 2 cardiologists, supported by a nurse, on a direct phone line is enough to ensure follow-up, and an AHF alert is easily analyzed when a patient record turns red on the telemonitoring system medical website. In the coming years, with a growing older population, simple methods of following older patients with HF will become necessary [15-18], and telemonitoring systems appear to be useful for that purpose, as proven in our study by the low rate of inaccurate measurements and the need for a landline only.

Another important finding of this study is that the device did not serve to improve the underlying treatment of CHF, but only to treat new-onset congestive HF as promptly as possible, to avoid long-stay hospitalizations and associated costs. This system is integrated in the overall management of patients with HF, providing daily monitoring and ready access to a cardiologist.

In our study, the median time from inclusion to the first AHF alert was 100 (IQR 36.00-205) days, with a shorter median time for unconfirmed AHF alerts than confirmed AHF alerts. This difference could be explained by the rate of inaccurate measurements in the unconfirmed AHF alert group; these inaccurate measurements seem to have occurred soon after inclusion because of misunderstanding or misuse, which are easily corrected by further instruction.

### Limitations

Our study has several limitations. First, a limited number of patients were included from a single center, which can lead to a lack of breadth for the study. Another limitation is the duration of this retrospective study: only 1 year. In fact, the telemonitoring system has actually been used in Nouvelle Clinique Tourangelle since 2017, but no study was conducted to check the efficacy of telemedicine for AHF. In a decade of rising use of telemedicine systems, they have come to play a part in HF monitoring after home discharge but should undergo a prospective clinical trial to statistically determine their effectiveness. Many clinical trials have proven the efficacy of telemedicine systems in reducing lengths of stay and rates of hospitalization [15,19,20]. The telemonitoring system should undergo a clinical trial to confirm its efficacy and possibly its ability to reduce length of hospital stay for AHF.

### Conclusion

Our study has shown the potential benefit in home health care of a telemonitoring system based on daily measurements of weight, TCOM, and cardiac frequency for quickly detecting and treating cardiac instability complicating the progression of CHF by detecting new-onset AHF, as well as new-onset supraventricular arrhythmia in ambulatory patients, thus helping cardiologists provide closer follow-up.

## Abbreviations

AHF: acute heart failure
bpm: beats per minute
CHF: chronic heart failure
ETAPES: Expérimentations de Télémédecine pour l’Amélioration des Parcours En Santé
HF: heart failure
HFpEF: heart failure with preserved ejection fraction
HFrEF: heart failure with reduced ejection fraction
HR: heart rate
LVEF: left ventricular ejection fraction
TCOM: transcutaneous oxygen measurement

## Data Availability

The data sets generated during and/or analyzed during this study are available from the corresponding author on reasonable request.

## References

[1] McDonagh Theresa A, Metra Marco, Adamo Marianna, Gardner Roy S, Baumbach Andreas, Böhm Michael, Burri Haran, Butler Javed, Čelutkienė Jelena, Chioncel Ovidiu, Cleland John G F, Coats Andrew J S, Crespo-Leiro Maria G, Farmakis Dimitrios, Gilard Martine, Heymans Stephane, Hoes Arno W, Jaarsma Tiny, Jankowska Ewa A, Lainscak Mitja, Lam Carolyn S P, Lyon Alexander R, McMurray John J V, Mebazaa Alexandre, Mindham Richard, Muneretto Claudio, Francesco Piepoli Massimo, Price Susanna, Rosano Giuseppe M C, Ruschitzka Frank, Kathrine Skibelund Anne (2021). 2021 ESC Guidelines for the diagnosis and treatment of acute and chronic heart failure. Eur Heart J.

[2] Scholte N, Gürgöze Muhammed T, Aydin D, Theuns D, Manintveld O, Ronner E, Boersma Eric, de Boer Rudolf A, van der Boon Robert M A, Brugts Jasper J (2023). Telemonitoring for heart failure: a meta-analysis. Eur Heart J.

[3] Steele Gray C, Tang T, Armas A, Backo-Shannon M, Harvey S, Kuluski K, Loganathan M, Nie JX, Petrie J, Ramsay T, Reid R, Thavorn K, Upshur R, Wodchis WP, Nelson M (2020). Building a digital bridge to support patient-centered care transitions from hospital to home for older adults with complex care needs: protocol for a co-design, implementation, and evaluation study. JMIR Res Protoc.

[4] Koehler F, Koehler K, Prescher S, Kirwan B, Wegscheider K, Vettorazzi E, Lezius S, Winkler S, Moeller V, Fiss G, Schleder J, Koehler M, Zugck C, Störk Stefan, Butter C, Prondzinsky R, Spethmann S, Angermann C, Stangl V, Halle M, von Haehling S, Dreger H, Stangl K, Deckwart O, Anker SD (2020). Mortality and morbidity 1 year after stopping a remote patient management intervention: extended follow-up results from the telemedical interventional management in patients with heart failure II (TIM-HF2) randomised trial. Lancet Digit Health.

[5] Roubille F, Mercier G, Lancman G, Pasche H, Alami S, Delval C, Bessou A, Vadel J, Rey A, Duret S, Abraham E, Chatellier G, Durand Zaleski I (2024). Weight telemonitoring of heart failure versus standard of care in a real-world setting: results on mortality and hospitalizations in a 6-month nationwide matched cohort study. Eur J Heart Fail.

[6] Galinier M, Roubille F, Berdague P, Brierre G, Cantie P, Dary P, Ferradou J, Fondard O, Labarre JP, Mansourati J, Picard F, Ricci J, Salvat M, Tartière Lamia, Ruidavets J, Bongard V, Delval C, Lancman G, Pasche H, Ramirez-Gil Juan Fernando, Pathak A, OSICAT Investigators (2020). Telemonitoring versus standard care in heart failure: a randomised multicentre trial. Eur J Heart Fail.

[7] Koehler F, Koehler K, Deckwart O, Prescher S, Wegscheider K, Kirwan B, Winkler S, Vettorazzi E, Bruch L, Oeff M, Zugck C, Doerr G, Naegele H, Störk Stefan, Butter C, Sechtem U, Angermann C, Gola G, Prondzinsky R, Edelmann F, Spethmann S, Schellong SM, Schulze PC, Bauersachs J, Wellge B, Schoebel C, Tajsic M, Dreger H, Anker SD, Stangl K (2018). Efficacy of telemedical interventional management in patients with heart failure (TIM-HF2): a randomised, controlled, parallel-group, unmasked trial. Lancet.

[8] Cleland JFG, Clark RA (2018). Telehealth: delivering high-quality care for heart failure. Lancet.

[9] Emani S (2017). Remote monitoring to reduce heart failure readmissions. Curr Heart Fail Rep.

[10] Jiménez-Marrero Santiago, Yun S, Cainzos-Achirica M, Enjuanes C, Garay A, Farre N, Verdú Jose M, Linas A, Ruiz P, Hidalgo E, Calero E, Comín-Colet Josep (2020). Impact of telemedicine on the clinical outcomes and healthcare costs of patients with chronic heart failure and mid-range or preserved ejection fraction managed in a multidisciplinary chronic heart failure programme: A sub-analysis of the iCOR randomized trial. J Telemed Telecare.

[11] Louis AA, Turner T, Gretton M, Baksh A, Cleland JG (2003). A systematic review of telemonitoring for the management of heart failure. Eur J Heart Fail.

[12] Radhakrishnan K, Jacelon C (2012). Impact of telehealth on patient self-management of heart failure: a review of literature. J Cardiovasc Nurs.

[13] Grady K (2008). Self-care and quality of life outcomes in heart failure patients. J Cardiovasc Nurs.

[14] Ding H, Jayasena R, Chen SH, Maiorana A, Dowling A, Layland J, Good N, Karunanithi M, Edwards I (2020). The effects of telemonitoring on patient compliance with self-management recommendations and outcomes of the innovative telemonitoring enhanced care program for chronic heart failure: randomized controlled trial. J Med Internet Res.

[15] Kotb A, Cameron C, Hsieh S, Wells G (2015). Comparative effectiveness of different forms of telemedicine for individuals with heart failure (HF): a systematic review and network meta-analysis. PLoS One.

[16] Brignell M, Wootton R, Gray L (2007). The application of telemedicine to geriatric medicine. Age Ageing.

[17] Di Lenarda Andrea, Casolo Giancarlo, Gulizia Michele Massimo, Aspromonte Nadia, Scalvini Simonetta, Mortara Andrea, Alunni Gianfranco, Ricci Renato Pietro, Mantovan Roberto, Russo Giancarmine, Gensini Gian Franco, Romeo Francesco (2016). [ANMCO/SIC/SIT Consensus document: The future of telemedicine in heart failure]. G Ital Cardiol (Rome).

[18] Chen SH, Edwards I, Jayasena R, Ding H, Karunanithi M, Dowling A, Layland J, Maiorana A (2021). Patient perspectives on innovative telemonitoring enhanced care program for chronic heart failure (ITEC-CHF): usability study. JMIR Cardio.

[19] Lin M, Yuan W, Huang T, Zhang H, Mai J, Wang J (2017). Clinical effectiveness of telemedicine for chronic heart failure: a systematic review and meta-analysis. J Investig Med.

[20] Zhu Y, Gu X, Xu C (2020). Effectiveness of telemedicine systems for adults with heart failure: a meta-analysis of randomized controlled trials. Heart Fail Rev.

